# Adapt, Explore, or Keep Going? The Role of Adaptability, Curiosity, and Perseverance in a Network of Study-Related Factors and Scholastic Success

**DOI:** 10.3390/jintelligence11020034

**Published:** 2023-02-13

**Authors:** Tommaso Feraco, Enrico Sella, Chiara Meneghetti, Giorgia Cona

**Affiliations:** Department of General Psychology, University of Padova, 35122 Padova, Italy

**Keywords:** 21st century skills, adaptability, grit, curiosity, academic achievement, self-regulated learning, motivation, achievement emotions, life satisfaction, network analysis

## Abstract

Soft skills are the key characteristics for students’ success and wellbeing in the 21st century, but they were only rarely studied contemporarily or integrated into comprehensive models of self-regulated learning. This makes it difficult to understand the role that specific skills have above and beyond the others and how they work together to favor students’ achievement and life satisfaction. For this reason, in a sample of 585 students (10–18 years old), we applied an exploratory network analysis and studied three crucial soft skills (i.e., adaptability, curiosity, and perseverance) and their contemporary network of inter-relationships with a host of functional study-related factors, including self-regulated learning strategies, motivation, emotions, cognitive abilities, academic achievement, and life and school satisfaction. Results show that the three soft skills play a positive role within the school context through their association with the majority of the study-related factors that mediate their relationships with academic achievement. Importantly, the results differentiated adaptability (which mainly relates with wellbeing and emotional variables), perseverance (which relates with the cognitive and behavioral aspect of learning), and curiosity (which bridges the connection between the other skills and relates with emotional and behavioral variables) in the school context. Overall, these findings contribute to the deepening of the theoretical framework on soft skills and their role as part of a successful learning profile, and inform us about the possible effectiveness of intervention on soft skills for students’ achievement and wellbeing.

## 1. Introduction

National and international agencies (European Commission 2016; Greiff and Borgonovi 2022; Robles 2012; World Economic Forum 2016) are increasingly focusing on the research and development of soft skills to equip youths with competencies expendable in different fields of their lives that might also be useful in facing 21st century challenges (including climate change, cultural transformations, or evolving technologies) and other challenging everyday-life situations. Among the different frameworks and taxonomies of soft skills, adaptability, curiosity, and perseverance (defined below) appeared frequently and are considered able to impact people’s success (World Economic Forum 2016), especially in school. They, indeed, consistently are shown to be the three crucial aspects for students’ success and wellbeing at various school ages. Indeed, extensive literature deepened their role in the school context (Credé et al. 2017; Martin et al. 2017; von Stumm et al. 2011), and in relation to key study-related factors (e.g., academic self-efficacy) and students’ life satisfaction (Feraco et al. 2022a). Importantly, soft skills, such as perseverance, curiosity, or adaptability, sustained students during the COVID-19 pandemic (Feraco et al. 2022b; Martin et al. 2021; Stockinger et al. 2021; Sulla et al. 2022). In this case, they favored students’ emotions, their adaptation to new learning modalities, and academic performance, among others. Nonetheless, they have generally been studied separately, or as part of a single factor (Feraco et al. 2022a) and a comprehensive analysis of their specific and contemporary role within the school context is still missing. In other words, less is known about the effect of each skill on academic achievement and study-related factors when the other soft skills are taken into account. For this reason, we decided to adopt a network analysis approach, which is a new statistical approach for basic psychological research that can unveil the complex pattern of the inter-relationships of these skills with various study-related factors and scholastic success indices, including academic achievement and general and school-related satisfaction. This approach allows us to better understand the specificity of each soft skill within a comprehensive model of learning and their direct and mediated relations with academic achievement and general and school-related satisfaction. In particular, we might expect soft skills to have a mediated association with academic achievement (Feraco et al. 2023a) and adaptability, curiosity, and perseverance to have peculiar associations with the other factors. Adaptability, for example, might emerge as more directly associated with emotions compared to other skills (Zhang et al. 2021).

This approach is in line with recent models in self-regulated learning (SRL) research. These highlight the need of a broad focus on the inter-relationships between all the intra-individual characteristics that drive the individual’s trajectory through learning and development to better understand how these factors work together to sustain students’ learning and wellbeing (Ben-Eliyahu 2019; Ben-Eliyahu and Bernacki 2015). Within these models, soft skills are theoretically included together with other study-related factors, such as academic self-efficacy (i.e., beliefs about one’s own academic abilities in academic tasks; Bandura et al. 1997), learning goals (i.e., the tendency to approach study materials to expand ones’ knowledge and skills; Dweck and Leggett 1988), SRL strategies (i.e., behavioral and metacognitive strategies that students adopt through the entire learning cycle; Efklides 2011; Pintrich 2004), achievement emotions (i.e., the emotions directly related to learning activities and outcomes; Pekrun 2006), scholastic engagement (i.e., active and sustained involvement in behavioral scholastic aspects: participation, endeavor, and attention in class; Wang et al. 2019), and fluid reasoning, which is the strongest direct predictor of academic achievement (Roth et al. 2015) and is involved in thinking, memorizing, and processing information from study materials.

### 1.1. Soft Skills and Their Role at School

Soft skills are personal characteristics that help individuals efficiently regulate their emotions, thoughts, and behaviors to finally complete and succeed in a task (Feraco et al. 2022a; Park et al. 2004; Robles 2012). On this line, their importance in promoting academic performance and study-related factors, as well as sustaining life satisfaction and positive emotions at school, has increasingly been recognized (Feraco et al. 2023a; Graesser et al. 2022; Soto et al. 2022; Weber and Harzer 2022). Here, we will focus on three of them, namely, adaptability, curiosity, and perseverance, because, among the others, these are probably the most studied in the school context.

In particular, adaptability, or the ability to successfully regulate thoughts, emotions, and behaviors in front of new and uncertain situations (Martin et al. 2012), has been associated with higher levels of motivational factors, such as academic self-efficacy (Feraco et al. 2023b; Martin et al. 2017), growth mindset (Martin et al. 2012), mastery learning goals (Burns et al. 2018; Martin et al. 2017), positive achievement emotions (Feraco et al. 2023b; Zhang et al. 2021), scholastic engagement (Burns et al. 2018; Collie et al. 2017), and SRL strategies (Feraco et al. 2023b). This confirms the idea that students with higher adaptability levels are also more able to cope with the continuous changes that schools present them: different schools (e.g., moving from middle school to high school), different subjects, schoolmates, unexpected tests, changing teachers, and so on.

Likewise, curiosity, or the dispositional desire toward learning, exploring, and experiencing that drives people to acquire new information and knowledge (Berlyne 1960), has been labeled as the ‘*third pillar*’ of academic performance (von Stumm et al. 2011) for its importance in learning and achieving success at school. In particular, more curious students were shown to obtain higher success at school, in terms of academic achievement (Tang and Salmela-Aro 2021; von Stumm et al. 2011), to use deeper SRL strategies, and to be more motivated and engaged toward learning (Chan et al. 2012; Feraco et al. 2022a; Hulme et al. 2013; Richards et al. 2013).

The scenario is similar for perseverance, or the tendency to work hard toward a goal despite setbacks and difficulties (Duckworth et al. 2007), which has been longitudinally associated not only with students’ academic achievement (Jiang et al. 2019; Postigo et al. 2021), but also with self-efficacy and learning goals (Akin and Arslan 2014; Datu et al. 2017; Muenks et al. 2018), positive achievement emotions (Datu and Fong 2018; Feraco et al. 2023a), and SRL strategies (Muenks et al. 2017; Wolters and Hussain 2015). In other words, students who persist toward their learning goals are more motivated, select SRL strategies efficiently, and generally feel better emotions through the learning pattern.

The similarities between these soft skills (and others) recently led researchers to consider such specific learning skills as a unique second-order factor that, when included in a path analysis with various study-related factors, seemed to favor other key learning variables, such as students’ motivation, SRL strategies, achievement emotions, and life satisfaction, indirectly affecting academic achievement (Feraco et al. 2022a, 2023a). This also confirms the idea that soft skills, in general, sustain the individuals in efficiently regulating their emotions, thoughts, and behaviors at school (Feraco et al. 2022a; Park et al. 2004; Robles 2012). Nonetheless, does each soft skill have a different pattern of relationships with study-related factors above and beyond the other soft skills? The answer to this question is not clear yet. Taking a different approach (i.e., a network approach), we might better frame it. Indeed, an exploratory network approach allows us to deepen the complex pattern of the inter-relationships between soft skills and study-related factors in the school context. In particular, the network approach can highlight the specificity of each soft skill for study-related and success factors. In other words, in order to capture the specific role of soft skills, it might be better to conceive the single soft skills and study-related factors as parts of a large network of inter-relationships in which each variable represents a node that is connected to the others through a more complex pattern of direct and indirect associations.

### 1.2. Exploring Soft Skills and Their Complex Role through Network Psychometrics Modeling

Under this light, we aim to expand the knowledge about the role of three crucial soft skills (i.e., adaptability, curiosity, and perseverance) in the school context by accompanying previous confirmatory results with a more explorative network analysis that might better inform us about their specificities within a network of functional scholastic variables.

A network approach makes things a bit more complex because it is generally based on a larger number of variables than usual, it does not consider linear causal relationships between two variables but works on the totality of the associations between the variables, and it is generally data driven, making it implausible to propose a priori hypotheses and follow strict psychological theories. Nonetheless, it has its advantages because psychological variables rarely work in the vacuum of simple directional regression analysis. On the contrary, psychological variables are inter-related in a wide network of causes, effects, and mutual relationships (Modafferi et al. 2022), which can only be detected with a more holistic approach to the data, such as network analysis (Borsboom et al. 2021; Costantini et al. 2015; Dalege et al. 2017; Sporns 2011). Importantly, one approach should not be preferred to the other since they have different aims and advantages, and both are valid but can give different hints. Regression analysis allows to test specific theories and informs us about the importance of a construct, possibly driving to interventions on a construct (e.g., adaptability) to promote a desirable outcome (e.g., life satisfaction); network analysis, on the other side, informs about the specificity of that construct within a complex network of functional associations, thus expanding its nomological network and deepening the pathway of causal relationships connecting one construct to another, which is the aim of this study.

Another interesting feature of network analysis is centrality indices, which inform us about the importance of each node within the network. The most common indices are the *strength* of a node, or the sum of all the values associated with that node; the *betweenness*, or how many times a node lies within the shortest path connecting two other nodes of the network; the *closeness*, or how close a node is to all the other nodes of the network; and the *expected influence*, or how much changing the value of a node influences the other nodes of the network. We could practically think of centrality indices as information about how much an intervention on a specific node can affect the other variables considered, in absolute terms. Intervening on a node with high *closeness* will possibly affect many other nodes (at different degrees). Differently, an intervention on a node with higher *strength* could have an effect that is bigger in magnitude, but that potentially affects only a few variables.

### 1.3. Rationale of the Study

On the above premises, we aim to examine the inter-relationship between three key soft skills (i.e., adaptability, curiosity, and perseverance), study-related factors (SRL strategies, learning goals, academic self-efficacy, positive and negative achievement emotions, behavioral engagement, and fluid reasoning), academic achievement, and life and school satisfaction by means of an explorative network analysis. This approach might better inform us about soft skills’ specificities within a network of positive scholastic variables, thereby improving our understanding of their complex pattern of relationships and their different roles. In other words, a network analysis could shed light on the complex pattern of direct and indirect relations characterizing each specific soft skill. It also offers us the opportunity to calculate centrality indices that could be crucial for future interventions. In fact, they inform us about the strength and importance of each node within the network of associations, instead of focusing only on the significance or on the strength of bivariate associations.

In particular, starting from previous findings that link adaptability, curiosity, and perseverance with academic achievement, life satisfaction, and a host of positive study-related variables (e.g., Feraco et al. 2022a), we will explore a network comprehensive of:

#### 1.3.1. Positive Predictors of Scholastic Success

- *SRL strategies*, which are key aspects of learning and among the highest predictors of academic achievement (Richardson et al. 2012);
- *Motivational beliefs* (in terms of academic self-efficacy and learning goals), which drive the students to learn and approach study materials (Kriegbaum et al. 2018; Pintrich 2003) and have also been historically associated with higher success at school (Lavrijsen et al. 2022);
- *Achievement emotions*, which play an important role in learning as positive predictors of SRL strategies, motivation, and academic achievement (Forsblom et al. 2022; Huang 2012; Mega et al. 2014). We will separately consider negative and positive achievement emotions;
- *Scholastic engagement*, and behavioral scholastic engagement in particular (e.g., being on time, asking questions in class, completing homework, or contributing to learning activities). Only behavioral engagement was assessed because it is the one more associated with academic achievement (Lei et al. 2018) and because possible latent factors should be avoided in network analysis (Modafferi et al. 2022), making them impractical to include different facets of engagement;
- *Fluid reasoning*, which is involved in thinking, memorizing, and processing information from study materials. Despite its importance for academic achievement, it is generally unrelated or only slightly related with soft skills, SRL aspects, and life satisfaction (Feraco et al. 2023a; Kriegbaum et al. 2018; Lavrijsen et al. 2022; Lechner et al. 2022).

#### 1.3.2. Scholastic Success Outcomes

Scholastic success is a multi-componential outcome that does not encompass only academic achievement but also focuses on students’ competences and wellbeing (York et al. 2015). For this reason, and to have a more holistic picture of scholastic success and its relation with the other variables considered, we included:

- *Academic achievement*, in terms of scholastic grades obtained by students;
- *Students’ life satisfaction* (i.e., one’s contentment with own’s life; Diener et al. 1985);
- *Students’ school satisfaction*, or how much they are satisfied with their school life (Huebner 1994).

### 1.4. Hypotheses

Although network psychometric modeling is by nature exploratory, based on the above-mentioned literature and the theoretical definitions of the three specific soft skills, we expect that:

- In accordance with previous confirmatory studies (Feraco et al. 2022a, 2023a; Muenks et al. 2017), adaptability, curiosity, and perseverance are distal predictors of academic achievement and precursors of study-related factors; for this reason, we expect them not to be central nodes in the network and not to be directly associated with academic achievement. This hypothesis could be also strengthened by the inspection of centrality indices, which should be low in case soft skills are actually distal factors of learning.
- Despite their distance from academic achievement, adaptability, curiosity, and perseverance should play a positive role, albeit with varying degrees, within the network of association with other study-related factors and scholastic success variables. In particular, the following might be expected:
  ○ Adaptability could have a more direct role in regulating emotions compared to curiosity and perseverance. This is because it is specifically characterized by an emotional component (Martin et al. 2012);
  ○ Curiosity could be more strongly related to motivational beliefs and particularly learning goals because it entails the desire to expand one’s knowledge and skills (Berlyne 1960);
  ○ Perseverance could be particularly important for behavioral aspects of learning and engagement, as it describes one’s tendency to work hard toward one’s goals. Perseverance, however, does not directly entail regulating emotions and might have a less pronounced effect on emotional variables (Duckworth et al. 2007).

## 2. Materials and Methods

### 2.1. Participants

A total of 585 students (62% females) between 10 to 18 years old (M_age_ = 12.56, SD = 1.90) from 29 different classes voluntarily participated in the study after their parents (or the student if 18 years old) signed the consent form. 337 students were from middle schools (grades 6 to 8) while 266 were from high schools (grades 9 to 12). Schools were all public and distributed across three regions of Italy (i.e., Veneto, Piemonte, and Emilia Romagna). All the participants speak Italian fluently. Another 56 students participated in the study but did not complete all the tasks and were consequently not considered in the analysis. Part of the data used for this study, including study-related factors, academic achievement, and life satisfaction, but not the three soft skills and school satisfaction, were already published in a previous paper. The study was approved by the University Ethics Committee for Research in Psychology.

No rules for detecting the minimum sample size with network analysis are available at the moment (Epskamp et al. 2018); however, the sample size considered is in line with previous studies using network analysis with even more variables than we did (Modafferi et al. 2022; Tosi et al. 2020). We additionally ensured the stability of the network’s parameter using a bootstrap approach. This showed that the main parameters remain stable (i.e., correlate >0.70 with original parameters), also dropping between 16% and 57% of the sample.

### 2.2. Measures

All the materials included in the study showed good internal consistency and psychometric properties in the original validation articles and on the actual sample. Alpha and omega reliability coefficients calculated on our sample are reported in Table 1. All the questionnaires were administered in Italian. The persistence and the engagement scale were adapted in Italian for this study; all the other questionnaires were already validated in Italian.

#### 2.2.1. Soft Skills

Three questionnaires were used to measure the three soft skills of interest:

The *Adaptability Scale* (Martin et al. 2012; Italian version by Feraco et al. 2023b) measures the ability to efficiently regulate emotional, behavioral, and cognitive responses to new and uncertain situations. It includes nine items on a 7-point Likert scale (e.g., “I am able to adjust my thinking or expectations to assist me in a new situation”). The total score was calculated as the sum of all the items (Max: 56).

The *Epistemic Curiosity Scale* (Litman and Spielberger 2003; Italian version by Lauriola et al. 2015) measures the epistemic desire of learning and acquiring new information. It includes ten items on a 7-point Likert scale (e.g., “Enjoy learning about subjects which are unfamiliar”). The total score was calculated as the sum of all the items (Max: 70).

The *Perseverance scale* (adapted from Miller et al. 1996) measures the tendency toward completing a task despite difficulties and setbacks that might be encountered. It includes eight items (six are reverse-scored) on a 5-point Likert scale (e.g., “If I have trouble solving a problem, I’ll try to get someone else to solve it for me”). The total score was calculated after reversing the six negative items (Max: 40).

#### 2.2.2. Study-Related Factors

The *Self-Regulated Learning Questionnaire* (De Beni et al. 2014) measures the use of functional SRL strategies in terms of elaboration, strategies, metacognition, organization, and self-evaluation (e.g., “I understand when the task I have to do is easy or difficult for me.”). It includes 50 items on a 5-point Likert scale. The total score was calculated after reversing the negative items (Max: 250).

The *Motivation Questionnaire* (De Beni et al. 2014) was used to measure two aspects of scholastic motivation: academic self-efficacy (e.g., “How do you rate your study skills?”) and learning goals, in terms of mastery learning goals (e.g., “It’s more important to me to learn things than to get good grades.”). It includes nine items on a 5-point Likert scale: five items for the self-efficacy subscale and four items for the learning goals subscale. The total score was calculated for each subscale after reversing negative items (Max: 20).

The *Student Engagement Scale* (adapted from Fredricks et al. 2011) was used to measure behavioral engagement at school. It includes eight items on a 5-point Likert scale (e.g., “How often do you really pay attention during math classes?”). The total score was calculated for each subscale after reversing negative items (Max: 40).

The *Positive and Negative Affect Schedule* (Watson et al. 1988; Italian version by Terraciano et al. 2003) was used to measure positive and negative emotions at school. It includes ten items for negative emotions (e.g., “scared”) and ten items for positive emotions (e.g., “determined”). Items are on a 5-point Likert scale and students indicate how much they experienced each of the twenty emotions at school during the past two weeks. The total score for each of the two subscales was calculated (Max: 50).

The *Culture-Free Intelligence Test* (Cattell 1940) was used as a proxy of fluid reasoning. It consists of 46 items divided into four reasoning subtasks (series completion, odd-one-out, matrices, and topology). One point is awarded for each correct answer and the total score is calculated as the sum of all the subtasks’ scores (Max: 46).

#### 2.2.3. Scholastic and Satisfaction Outcomes

*Academic achievement.* Students’ school grades in four subjects (i.e., math, science, Italian, and English) that are transversal between middle and high schools were provided by the responsible teachers of the class. Grades are given on a 10-point scale, where 6 is a pass. The average grade was calculated as a measure of general academic achievement.

The *Satisfaction With Life Scale* (Diener et al. 1985; Italian version by Di Fabio and Gori 2016) was used to measure students’ overall life satisfaction. It includes five items on a 7-point Likert scale (e.g., “The conditions of my life are excellent.”). The total score was calculated.

The *School* subscale of the *Multidimensional Students’ Life Satisfaction Scale* (Huebner et al. 2012; Italian version by Zappulla et al. 2014) was used to measure students’ satisfaction with their school. It includes five items on a 4-point Likert scale (e.g., “I look forward to going to school”). The total score was calculated.

### 2.3. Procedure

The first author and three trained psychologists contacted the schools and scheduled a meeting with the schools’ responsible teachers to explain the project in detail. Schools were randomly selected from regions that were close to the experimenters’ domiciles for practical reasons. Consent forms were distributed to all the parents whose children were involved in the project. Once they returned the signed consent forms, two data collection days were scheduled with each class (50 min max each) and a trained psychologist—with the presence of the teacher—administered the tasks and the questionnaires in a balanced order. Students read the instructions of all proposed questionnaires before completing them. They were asked to give the answer which seems to better describe them. A psychologist was also present during the administration of the questionnaires to deal with any students’ doubts. For each subscale of the Culture-Free Intelligence Test, the experimenter read the corresponding instructions and completed the practice items together with the students to be sure that the students understood what they had to do in the following test items. When everyone understood the task’s request, the actual test began. At the end of the school semester, the responsible teachers provided school grades for each student involved. School grades were automatically merged with the other measures through an anonymized code.

### 2.4. Data Analysis

The analysis of the data were run in R (R Core Team 2020) and followed two steps.

#### 2.4.1. Reliability and Bivariate Correlations

First, we calculated all the reliability coefficients of the scales included in the study using the *reliability()* function of the ‘semTools’ package (Jorgensen et al. 2022). Then, we calculated their bivariate correlations. This step has a descriptive purpose of the associations between the variables considered, but it also allows a direct comparison with what was usually found in literature when studies focused on single soft skills and their associations with different scholastic and non-scholastic outcomes. Indeed, it should be expected that adaptability, curiosity, and perseverance are correlated with all the other variables considered, with the plausible exception of academic achievement (for adaptability) and fluid reasoning (for adaptability and perseverance).

#### 2.4.2. Network Analysis

Second, we ran a network analysis to unveil the network of associations between all the variables considered. Network analysis identifies a network of links between nodes (the variables of interest) that can be graphically depicted in an image representing nodes’ positions and the magnitude of their associations. To do so, partial correlations between all the variables are calculated and the LASSO algorithm applied (“least absolute shrinkage and selection operator”; Epskamp et al. 2018). LASSO regularization shrinks all the estimated correlations and set the lowest ones exactly to zero to avoid spurious correlations to interfere in the estimation of the parameters. This also facilitates the interpretation of the findings because a sparser, more readable, and more reliable graph will be estimated and depicted following this procedure (Costantini et al. 2015).

Other than edges’ weights (i.e., the strength of the associations), we also calculate centrality indices of the network: the *strength* of a node; its *betweenness*; its *closeness*; and its *expected influence*, or how much changing the value of a node influences the other nodes of the network. We will report and comment on these indices, but the focus of our analysis relies on the role of three specific nodes of the network (adaptability, curiosity, and perseverance) and this will remain the focus of our results and discussion. Additionally, previous studies using regression analysis placed the three soft skills as more distal predictors of academic achievement and could consequently be expected to be on the borders of a network of learning (Feraco et al. 2022a), thus having low centrality values.

## 3. Results

### 3.1. Bivariate Correlations

The results of the bivariate correlations (see Table 1 for the correlations of interest and Table A1 in Appendix A for the complete correlation matrix) are in line with what is found in literature (Feraco et al. 2022a). In particular, for what concerns adaptability, curiosity, and perseverance, it emerged that: adaptability is significantly (*p* < .001) correlated with all the other measures except reasoning and achievement (significant |*r*| ranging between 0.17 and 0.41); curiosity is significantly (*p* < .001) correlated with all the other measures except negative emotions, life satisfaction, reasoning, and achievement (significant |*r*| ranging between 0.23 and 0.41); and perseverance is significantly (*p* < .001) correlated with all the other measures except reasoning (significant |*r*| ranging between 0.19 and 0.54).

### 3.2. Network Results

**Figure 1:**
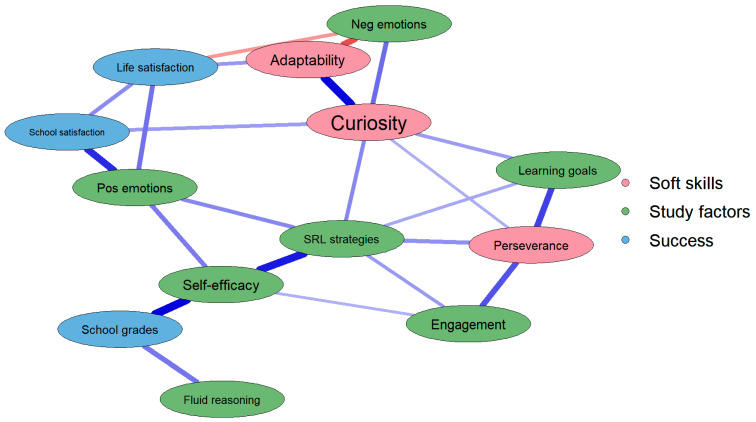
Results of the network analysis after LASSO regularization.

The results of the network analysis are depicted in Figure 1. In general, we can see that adaptability, curiosity, and perseverance play a distal role in academic achievement within the network of associations. At the same time, their specific role seems to be different in relation to study-related factors.

Adaptability resulted in a direct association not only with life satisfaction (*r_partial_* = 0.15) and negative achievement emotions (*r_partial_* = −0.25), but also with the second skill of interest: curiosity (*r_partial_* = 0.35). On its side, curiosity resulted in a positive association with negative achievement emotions (*r_partial_* = 0.20), mastery learning goals (*r_partial_* = 0.14), SRL strategies (*r_partial_* = 0.17), school satisfaction (*r_partial_* = 0.14), and perseverance (*r_partial_* = 0.12). Finally, perseverance resulted in a positive association with learning goals (*r_partial_* = 0.26), SRL stratgies (*r_partial_* = 0.16), and engagement (*r_partial_* = 0.24).

The only two nodes associated with academic achievement were fluid reasoning (*r_partial_* = 0.19) and self-efficacy (*r_partial_* = 0.35).

In general, the graph also shows associations between all the other variables and highlights a corner of the graph (top) that includes wellbeing variables intertwined with each other (i.e., life and school satisfaction and positive and negative emotions), and a corner (at the bottom) with more cognitive-behavioral aspects of learning that are again intertwined with each other (i.e., SRL strategies, learning goals, behavioral engagement, and self-efficacy).

#### Centrality Indices

**Figure 2:**
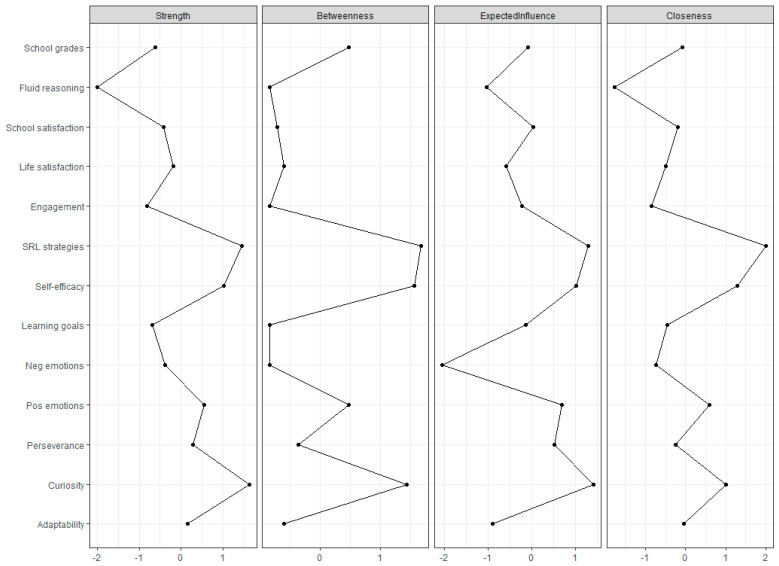
Centrality indices of the network.

Descriptively, we report in Figure 2 the results for the four indices explained in the statistical analysis section. As evident from the plots, SRL strategies, curiosity, and self-efficacy are constantly the three most influential nodes in the network. Fluid reasoning, on the contrary, has a small impact on this network. Nonetheless, it is one of the two direct predictors of academic achievement. Interestingly, the other two soft skills (i.e., adaptability and curiosity) are placed on the border of the model and their centrality indices are quite low, highlighting that their specificity only affects part of the network.

## 4. Discussion

Using an explorative network analysis, in this study, we examined the nomological framework of three soft skills, namely, adaptability, curiosity, and perseverance, in the school context. In particular, we assessed the specific influence of these three soft skills in a network structure comprehensive of crucial study-related factors and important outcome variables—such as life and school satisfaction, and academic achievement—in students between 10 to 18 years old.

Specifically, the network was based on 13 variables: adaptability, curiosity, and perseverance (i.e., the three soft skills), SRL strategies, behavioral engagement, positive and negative achievement emotions, self-efficacy, and learning goals (i.e., six key behavioral, emotional, and motivational factors of SRL), life and school satisfaction and academic achievement (i.e., proxies of students’ wellbeing and school success), and fluid reasoning abilities. Within the network of associations that emerged (Figure 1), we will focus our attention on the soft skills’ nodes and their roles within the SRL model (Ben-Eliyahu 2019) and for academic achievement and students’ satisfaction and emotions.

### 4.1. Adaptability, Curiosity, Perseverance, and Academic Achievement

Our results, in line with recent evidence (Feraco et al. 2023b; Muenks et al. 2017), showed that soft skills are distal from academic achievement, but highlighted their role as possible predictors of study-related factors that mediate the association between soft skills and academic achievement (Weber and Harzer 2022).

Firstly, it is important to mention that soft skills did not relate with fluid (reasoning) ability (in line with previous studies; Kretzschmar et al. 2022; Lechner et al. 2022), whereas SRL strategies represented the most central and influential node in the network. In short, these findings suggest that, rather than relating to fluid intelligence skills, the influence of soft skills in the school context may thus be directed towards the psychological and psychosocial variables of academic performance. Indeed, considering the three soft skills, our results showed that adaptability, curiosity, and perseverance are always close to the borders of the model and graphically distant from academic achievement, which in turn is directly connected to self-efficacy and fluid reasoning, and close to SRL strategies, positive achievement emotions, and engagement. These factors, as expected, lay on the paths connecting soft skills and academic achievement (e.g., curiosity → SRL strategies → self-efficacy → academic achievement). In other words, a curious student will benefit (academically) from curiosity only if it leads them to organize their study time in order to gain knowledge. Otherwise, if being curious leads to searching interesting but not pertinent information, the student will not gain (academically) from his/her curiosity. The distal role of soft skills for academic achievement is also highlighted by the low centrality indices associated with soft skills. These highlight that each soft skill is predominantly important for specific nodes of the network and not for the entire pattern of inter-relationships. Considering each soft skill individually within the network allowed us to understand how each one is differently involved in the network of relations, as reported in the paragraph below.

### 4.2. Specifics Roles of Adaptability, Curiosity, and Perseverance within a Network of Positive Learning Factors

Noteworthy findings emerged when we analyzed the complex structure of relationships between the three soft skills and study-related factors and scholastic success factors (such as life satisfaction and academic achievement) in a network framework. What is important to note for our aims is that, in line with our second hypothesis, the network model was able to highlight different peculiar roles for each of the three soft skills.

*Curiosity.* Curiosity is the only soft skill that shows a central role in the network (as is also descriptively found with the centrality indices) that associates it with half of the other nodes. Curious students, in fact, show better strategic and motivational beliefs of learning—as highlighted by the positive associations with SRL strategies and mastery learning goals—but also contemporarily show higher satisfaction with school and higher negative achievement emotions. In other words, curious students approach their study materials to enlarge their knowledge and skills and spend more time trying to organize their study materials, evaluate their learning, and select the best learning strategies (Chan et al. 2012; Richards et al. 2013), all behaviors and motivations that are functional for learning and academic achievement (Huang 2011; Richardson et al. 2012). At the same time, our findings highlight that these students enjoy their school time, like to learn new things at school, and the activities that their schools propose (i.e., they have higher school satisfaction) (Weber and Harzer 2022). However, curious students also feel more negative emotions at school. The latter is not totally unexpected. Curiosity, in fact, can be distinguished in “wanting” and “liking” new information (Litman 2005; Litman and Spielberger 2003), two drivers of curiosity that underlie different needs and emotions: on one hand, curious people like to obtain new information and enjoy learning, but on the other one, they sometimes need but cannot access (e.g., they cannot solve a riddle and cannot stop thinking about it; the teacher does not provide satisfying information; or activities are not always stimulating) new information. This might be frustrating for them and explain negative emotions at school in curious students (Kashdan et al. 2018). In addition, curiosity is linked to negative emotions also through the mediation of adaptability. The network, in fact, further suggests that students higher in both curiosity and adaptability might be able to find better emotional responses to these situations of uncertainty and eventually lower their negative emotions. Curiosity is also related to perseverance, thus assuming a central role in the connection between the three soft skills considered and suggesting that more curious people also tend to persevere more toward their goals, possibly driven by the deprivation pattern introduced above (Litman 2005).

*Perseverance.* Perseverance, on its side, emerges in the network as predominantly associated with the behavioral and motivational aspects of learning, but not with the emotional and wellbeing variables. In other words, perseverance—when controlling for all the other variables—has a much more specific role compared to what is found in the literature (Dametto and Noronha 2020; Feraco et al. 2023a; Weber and Harzer 2022). Students with higher perseverance will adopt SRL strategies to obtain their goals, will be more engaged (e.g., they will do their homework, and manage to be on time at school), and will set functional learning goals to increase their success chances but, contrary to previous findings, perseverance will not sustain their satisfaction nor their emotions at school, based on our data and analysis. This could be because perseverance is goal-oriented and students with higher levels of perseverance could persevere toward achieving academic success, regardless of their school and life satisfaction, or emotions.

*Adaptability.* Adaptability, on the opposite side, seems to intervene where perseverance does not: it directly relates with life satisfaction and negative emotions but has no direct connections with behavioral and motivational SRL aspects. Although these findings seemed to contradict previous evidence (Martin et al. 2012, 2017), it should be considered anyway that what we are referring to as adaptability here is only its very specific variance, while we know that it shares significant variance with the other soft skills and especially perseverance (Datu et al. 2017). It might probably be that it is this common variance that explains its association with the behavioral and motivational aspects of study-related aspects, as suggested by studies on the general factor of soft skills (e.g., Feraco et al. 2022a). Interestingly, the main role that adaptability assumed in the network is linked with achievement emotions, an effect that only recently, and mainly because of the stressful COVID-19 pandemic, captured the attention of scholars (Feraco et al. 2023b; Zhang et al. 2021). Future studies should then better understand the mechanism underlying the association between adaptability and emotions.

### 4.3. The Importance of Network Analysis

The results described are intriguing from a theoretical and practical perspective, and the adoption of a network approach allowed us to unveil the complex pattern of relationships between the variables of interest and individuate the specific roles that adaptability, curiosity, and perseverance play in students’ learning and wellbeing. Indeed, adopting this approach was crucial for different reasons: (a) we were able to confirm previous findings about the importance of soft skills for students using a different and exploratory approach; (b) we were also able to better delineate the nomological network of adaptability, curiosity, and perseverance within a single intra-individual SRL model (Ben-Eliyahu 2019) by studying their specific contemporary effects on all the variables of interest. This, with different approaches, would have requested a large number of regression analyses that, additionally, do not have the advantage of the LASSO regularization to deal with spurious and negligible correlations; and (c) we were also able to graphically represent the results in a readable and straightforward way. Importantly, these results not only can be well integrated with previous literature (e.g., Feraco et al. 2022a; Weber and Harzer 2022), but also advance our understanding about the peculiar role that each soft skill specifically has above their mutual and overlapping strengths with other important school-related variables.

### 4.4. Practical Implications of Network Modeling Approach in the School Context

The advantages of network analysis also reside on the practical side. In fact, one of the key aspects of network analysis is that it highlights the pattern of associations among the variables. In addition, it can potentially inform us on what can be affected more when intervening on a specific node. For this reason, network analysis is often used in studies of psychopathology (Borsboom 2017; Olthof et al. 2020). In our case, it can inform practitioners interested in supporting students’ learning or wellbeing about which characteristics should be promoted to benefit learning or wellbeing. Indeed, from the network that emerged from our data, it seems that sustaining students’ adaptability might have a stronger effect on the emotional and wellbeing aspects, but less on the motivational and behavioral advantages in learning. On the other side, if learning and SRL are our focus, fostering perseverance could be a better idea to indirectly promote different aspects of learning, such as SRL strategies, engagement, and learning goals. On the contrary, working on students’ curiosity could have a widespread effect (highlighted also by the centrality indices) on students’ satisfaction with their school and on their approach to learning, as highlighted by its direct relationship with SRL strategies and learning goals.

### 4.5. Limitations of the Study

Despite the novelty of the study and its methodological soundness, our work has some limitations that should be kept in mind. First, it only considers three soft skills among the many presented in literature. Their selection, however, was based on the importance that adaptability, curiosity, and perseverance gained in previous literature for what concerns students’ academic achievement. Nonetheless, future studies might consider integrating additional skills. Similarly, other school-related variables could be considered, including social variables such as relationships with peers or teachers. Additionally, the cross-sectional structure of the data prevented us from providing any additional and causal implications on the reciprocal influence between soft skills and the other variables considered here. It should be noted, however, that network analyses are by nature cross-sectional (even if longitudinal analyses are possible) and test patterns of mutual relationships and not causal relationships. It should also be noted that measures that are not only self-reported could increase the precision of the measurement; for example, engagement could be captured by observational methods of mixed methods (Fredricks 2022). Future studies could consider multiple and more ecological measurement methods to obtain more precise and reliable estimates.

## 5. Conclusions

Our findings integrate knowledge about soft skills’ role in schools by using a network approach to deepen their association with a host of positive learning and success factors. All the three soft skills considered (i.e., adaptability, curiosity, and perseverance) resulted in a positive integration within the network, but also showed specific patterns of associations that were not detectable with other methodological approaches. In particular, adaptability was shown to be more important than other skills for students’ life satisfaction and achievement emotions; perseverance was shown to be more important for what concerns behavioral and motivational aspects of learning; and curiosity was shown to have a bridging role between the other skills and between the emotional and behavioral/motivational component of school learning and success.

To conclude, our results show that a network approach can illuminate and enlarge previous findings by (a) highlighting the complex pattern of relationships occurring between psychological variables and (b) unveiling the specificity of individual soft skills in a large intra-individual model of SRL and school success.

## Figures and Tables

**Table 1:** Descriptive statistics and reliability indices (alpha and omega) of all the measures and correlations between soft skills and all the other variables.

	M (SD)	α	ω	Adaptability	Curiosity	Perseverance
Adaptability	4.56 (0.96)	0.77	0.79	-		
Curiosity	4.72 (1.04)	0.77	0.81	0.41 *	-	
Perseverance	3.58 (0.77)	0.82	0.83	0.23 *	0.36 *	-
Positive emotions	3.17 (0.72)	0.85	0.87	0.32 *	0.24 *	0.41 *
Negative emotions	2.25 (0.82)	0.87	0.89	−0.28 *	0.05	−0.19 *
Learning Goals	3.43 (0.83)	0.63	0.67	0.27 *	0.35 *	0.48 *
Self-efficacy	3.70 (0.64)	0.80	0.83	0.25 *	0.26 *	0.47 *
SRL strategies	3.36 (0.39)	0.85	0.87	0.28 *	0.40 *	0.54 *
Engagement	4.08 (0.53)	0.70	0.73	0.17 *	0.23 *	0.49 *
Life satisfaction	4.61 (1.29)	0.83	0.85	0.29 *	0.09	0.27 *
School satisfaction	2.64 (0.60)	0.78	0.82	0.25 *	0.33 *	0.42 *
Fluid Reasoning	0.70 (0.11)	0.61	0.62	0.06	0.06	0.11
School grades	7.09 (1.09)	-	-	0.04	0.07	0.32 *
Age	12.56 (1.90)	-	-	0.03	0.05	−0.15 *
Gender (F)	-	-	-	−0.14 *	0.08	0.00

* = *p* < .001. Mean and standard deviations refer to the average response for each item. Exceptions are school grades and age.

## Data Availability

Data are available on Figshare at the following link: https://10.6084/m9.figshare.21657350.

## Appendix A

**Table A1:** Reliability indices (alpha and omega) and bivariate correlations between study variables.

	α	ω	1.	2.	3.	4.	5.	6.	7.	8.	9.	10.	11.	12.	13.	14.
1.Adaptability	0.77	0.79	-													
2.Curiosity	0.77	0.81	0.41 *	-												
3.Perseverance	0.82	0.83	0.23 *	0.36 *	-											
4.Positive emotions	0.85	0.87	0.32 *	0.24 *	0.41 *	-										
5.Negative emotions	0.87	0.89	−0.28 *	0.05	−0.19 *	−0.17 *	-									
6.Learning goals	0.63	0.67	0.27 *	0.35 *	0.48 *	0.28 *	−0.19 *	-								
7.Self-efficacy	0.80	0.83	0.25 *	0.26 *	0.47 *	0.48 *	−0.21 *	0.31 *	-							
8.SRL strategies	0.85	0.87	0.28 *	0.40 *	0.54 *	0.51 *	−0.12	0.41 *	0.59 *	-						
9.Engagement	0.70	0.73	0.17 *	0.23 *	0.49 *	0.40 *	−0.15 *	0.23 *	0.45 *	0.47 *	-					
10.Life satisfaction	0.83	0.85	0.29 *	0.09	0.27 *	0.44 *	−0.28 *	0.20 *	0.34 *	0.29 *	0.31 *	-				
11.School satisfaction	0.78	0.82	0.25 *	0.33 *	0.42 *	0.53 *	−0.10	0.33 *	0.33 *	0.44 *	0.38 *	0.38 *	-			
12.Fluid reasoning	0.61	0.62	0.06	0.06	0.11	−0.02	−0.07	00.06	0.16 *	0.10	0.11	0.00	0.00	-		
13.School grades	-	-	0.04	0.07	0.32 *	0.18 *	−0.2 *	0.22 *	0.49 *	0.28 *	0.28 *	0.21 *	0.16 *	0.25 *	-	
14.Age	-	-	0.03	0.05	−0.15 *	−0.13	0.17 *	−0.08	−0.04	0.07	−0.10	−0.19 *	−0.10	0.17 *	−0.34 *	-
15.Gender (F)	-	-	−0.14 *	0.08	0.00	−0.11	0.28 *	−0.04	0.03	0.13	0.04	−0.16 *	0.11	0.12	−0.03	0.29 *

* = *p* < .001.
